# Supplementary material for: Diagnostic performance of the TR Chagas Bio-Manguinhos rapid test for detecting anti-Trypanosoma cruzi IgG in human samples from three Southern Cone countries
Source: PLoS Negl Trop Dis. 2026 May 8;20(5):e0014310. doi: 10.1371/journal.pntd.0014310 (PMC13178965; doi:10.1371/journal.pntd.0014310)
Supplement: Attachment — Submitted filename: Response to Reviewers.pdf [file pntd.0014310.s004.pdf]

Ministério da Saúde

FIOCRUZ
Fundação Oswaldo Cruz
Instituto Gonçalo Moniz

Salvador, Brazil - April 2026

Dear Editors,
PLos Neglected Tropical Diseases

We thank you and the reviewer for the careful evaluation of our revised manuscript and for the additional comment provided. In response, we have made the requested revisions and further clarified the implications of excluding discordant samples, particularly with respect to diagnostic performance estimates and generalizability. Detailed responses are provided below (Manuscript ID: PNTD-D-26-00016).

Thank you for your time and consideration.

Sincerely,

Fred Luciano Neves Santos
Corresponding author
fred.santos@fiocruz.br

Reviewer #2

Reviewer #2 – Question 1: The authors have addressed the comments well, and the manuscript has clearly improved. I recommend minor revisions. I encourage the authors to further expand the discussion on the exclusion of discordant samples, particularly regarding its potential impact on diagnostic performance estimates and generalizability.

Reply: We appreciate this important suggestion and have expanded the Discussion accordingly. Specifically, we now clarify that the exclusion of samples with discordant conventional serological results was a deliberate design choice consistent with the analytical objective of evaluating the index test in a well-characterized panel with a defined serological classification. We also explicitly acknowledge that, although this approach reduces uncertainty in the reference classification, it may have led to slightly optimistic estimates of diagnostic performance because diagnostically challenging samples that are more likely to occur in real-world screening algorithms were not represented. Accordingly, we now state that the present findings should be interpreted primarily as evidence of analytical performance in a well-defined retrospective panel, and that caution is warranted when extrapolating these estimates to broader programmatic settings in which inconclusive or discrepant serological profiles are part of routine practice. This point has also been reflected in the Methods section, where we now clarify the rationale for excluding discordant samples from the study panel.

New sentence incorporated to Discussion section:

"An additional limitation concerns the exclusion of samples with discordant conventional serological results from the study panel. This decision was deliberate and consistent with the analytical objective of evaluating the index test in a well-characterized sample set with a defined serological classification, as recommended in diagnostic validation studies [15]. Excluding discordant samples reduces uncertainty in the reference classification and helps avoid distortion of performance estimates caused by samples with unresolved infection status. However, this approach may also have led to slightly optimistic estimates of diagnostic performance, particularly because diagnostically challenging samples that are more likely to occur in real-world screening algorithms were not represented. Consequently, the present findings should be interpreted primarily as evidence of analytical performance in a well-defined retrospective panel, and caution is warranted when extrapolating these estimates to broader programmatic settings in which inconclusive or discrepant serological profiles are part of routine practice."

This point has also been reflected in the Methods section, where we now clarify the rationale for excluding discordant samples from the study panel.

Before: "…was used in routine practice; however, discordant cases were not included in the sample panel analyzed in the present study. The reference classification was based…"

After: …was used in routine practice; however, discordant cases were not included in the sample panel analyzed in the present study because the aim was to evaluate the index test in a panel with a defined serological classification. The reference classification was based…
