# Supplementary material for: Diagnostic performance of the TR Chagas Bio-Manguinhos rapid test for detecting anti-Trypanosoma cruzi IgG in human samples from three Southern Cone countries
Source: PLoS Negl Trop Dis. 2026 May 8;20(5):e0014310. doi: 10.1371/journal.pntd.0014310 (PMC13178965; doi:10.1371/journal.pntd.0014310)
Supplement: Attachment — Submitted filename: Response to Reviewers.docx [file pntd.0014310.s003.docx]

Salvador, Brazil - April 2026

**Dear Editors,**

PLos Neglected Tropical Diseases

We would like to express our sincere gratitude to you and to the reviewers for the thoughtful and constructive recommendations provided. The feedback has been invaluable in improving the quality and clarity of our manuscript. We believe that the revised version now presents a more accurate and comprehensive account of our research.

We are confident that this version aligns with the high standards of Plos Neglected Tropical Diseases, and we kindly request your consideration for publication. For ease of reference, we have provided detailed responses to the reviewers’ comments below (Manuscript ID: PNTD-D-26-00016).

Thank you very much for your attention and consideration.

Sincerely,

Fred Luciano Neves Santos

Corresponding author

fred.santos@fiocruz.br

**Editors' Recommendations**

**Journal Requirements. Potential Copyright Issues – Question 1:** Figure 1. Please (a) provide a direct link to the base layer of the map (i.e., the country or region border shape) and ensure this is also included in the figure legend; and (b) provide a link to the terms of use / license information for the base layer image or shapefile. We cannot publish proprietary or copyrighted maps (e.g. Google Maps, Mapquest) and the terms of use for your map base layer must be compatible with our CC BY 4.0 license. Note: if you created the map in a software program like R or ArcGIS, please locate and indicate the source of the basemap shapefile onto which data has been plotted. If your map was obtained from a copyrighted source, please amend the figure so that the base map used is from an openly available source. Alternatively, please provide explicit written permission from the copyright holder granting you the right to publish the material under our CC BY 4.0 license.

**Reply**: We thank the editor for this important clarification. The base map used in Figure 1 was obtained from the geoBoundaries cartographic database, an openly available resource of political administrative boundaries distributed under the CC BY 4.0 license. We have added the direct link to the source and clarified the licensing information in the figure legend. This source is compatible with the journal’s CC BY 4.0 requirements.

**“Fig 1. Flowchart of the study design in accordance with the Standards for Reporting of Diagnostic Accuracy Studies (STARD) guidelines.** The base map was obtained from the geoBoundaries cartographic database in shapefile format (.shp) (www.geoboundaries.org), an open database of political administrative boundaries distributed under the CC BY 4.0 license, and was subsequently reformatted and analyzed using QGIS version 3.10 (Geographic Information System, Open-Source Geospatial Foundation Project. http://qgis.osgeo.org).”

**Journal Requirements. Question 2:** Please ensure that the funders and grant numbers match between the Financial Disclosure field and the Funding Information tab in your submission form. Note that the funders must be provided in the same order in both places as well.

**Reply:** We thank the editor for this important clarification. We will carefully check the funders, grant numbers, and their order in both the Financial Disclosure field and the Funding Information tab before resubmission to ensure full consistency.

**Reviewer #1**

**Methods (Reviewer #1) – Study Design and Samples – Question 1:** Please indicate whether the study protocol is publicly available or can be shared upon request. If applicable, stating this explicitly would enhance transparency.

**Reply:** We thank the reviewer for this suggestion. This study was conducted using a retrospective design based on pre-existing samples from a well-characterized biorepository. Although a formal study protocol was not prospectively registered, all procedures followed established international recommendations for the serological diagnosis of chronic *Trypanosoma cruzi* infection, and all assays were performed strictly according to the manufacturers’ instructions. The manuscript has been revised according to the reviewer’s suggestion to ensure transparency and reproducibility.

**Methods (Reviewer #1) – Study Design and Samples – Question 2:** Please specify when and where the samples were collected (study sites and months/years), as well as the time interval between the index test and the reference standard.

**Reply:** We thank the reviewer for this important comment. We have clarified in the Methods section when and where the samples were collected, as well as the relationship between the reference standard and the index test. Samples were obtained from individuals evaluated at the Instituto Nacional de Parasitología “Dr. Mario Fatala Chabén” and at the Hospital General de Agudos “Dr. Ignacio Pirovano,” both in Buenos Aires, Argentina. Recruitment occurred between 2015 and 2024. The reference classification was based on serological testing performed in the clinical setting according to national and international recommendations, generally at the time of the initial evaluation or blood collection. In contrast, the TR Chagas Bio-Manguinhos assay was performed later on archived plasma samples. Because this was a retrospective study based on repository material, the time interval between the original reference testing and the index test was not standardized and may have varied across samples. This limitation has now been acknowledged in the manuscript.

**Methods (Reviewer #1) – Study Design and Samples – Question 3:** Given that samples were stored for several months or years, please clarify how sample quality was ensured. Were samples re-tested using the reference standard, or were reference results derived from the original study? A brief discussion of how this may influence interpretation of the results would be helpful.

**Reply:** We thank the reviewer for this important comment. Sample quality was preserved by storing plasma aliquots at –20 °C in multiple tubes, thereby minimizing repeated freeze–thaw cycles. The reference classification used in this study was based on the original serological results obtained in the hospital and institute settings and was not re-established by repeating all reference assays specifically for the present study. In addition, samples were re-evaluated in our laboratory using an in-house ELISA based on *T. cruzi* lysate as an additional internal characterization step. We acknowledge that the retrospective use of stored samples may introduce some uncertainty related to long-term storage and to the non-standardized interval between the original reference testing and the index test. This point has now been addressed in the Discussion to improve the interpretation of the findings.

**Methods (Reviewer #1) – Study Design and Samples – Question 4:** Please clarify whether commercial assays (eg. ELISA, HAI) or in-house methods were used as part of the composite reference standard, and include references as appropriate.

**Reply:** We thank the reviewer for this important comment. We have clarified in the Methods section that the composite reference classification used in this study was based on the original serological results obtained at the participating institutions using commercial assays routinely employed for the diagnosis of chronic T. cruzi infection. At the Instituto Nacional de Parasitología “Dr. Mario Fatala Chabén”, the diagnostic workup included CMIA Architect Chagas (ARCHITECT Chagas®, Abbott, Illinois, USA) and Chagatest ELISA recombinante v.4.0 (Wiener lab®, Rosario, Argentina). At the Hospital General de Agudos “Dr. Ignacio Pirovano”, the routine diagnostic assays included Chagatest HAI (Wiener lab®, Rosario, Argentina) and CMIA on the Alinity i platform (Abbott, Illinois, USA). In cases of discrepancy at Pirovano, an additional hemagglutination assay (SERODIA-CHAGAS, Fujirebio Inc., Tokyo, Japan) was used in routine practice; however, discordant cases were not included in the sample panel analyzed in the present study. The in-house ELISA based on *T. cruzi* lysate, performed in our laboratory, was not part of the composite reference standard and was used only as an additional internal characterization step. The manuscript has been revised according to the reviewer’s suggestion to ensure transparency and reproducibility.

**Methods (Reviewer #1) – Study Design and Samples – Question 5:** The sample size estimation would benefit from additional clarification. Please specify the power used, whether the reported margin of error (1.7) corresponds to a half-width or full-width interval, and provide a reference for the formula applied.

**Reply:** We thank the reviewer for this important comment. We have clarified in the Methods section that the reported margin of error (1.7%) corresponds to the half-width of the 95% confidence interval for sensitivity and specificity estimates. Sample size estimation was based on standard formulas for proportions in diagnostic accuracy studies, assuming expected sensitivity of 99% and specificity of 99.5% at a 95% confidence level. As this was a retrospective study using samples from an established biobank, a formal prospective power calculation was not applied. Nevertheless, the final sample size exceeded the minimum requirement for overall diagnostic performance estimation, particularly for sensitivity, which was the primary outcome of interest. An appropriate methodological reference has also been added (Zhou et al., 2011). The manuscript has been revised according to the reviewer’s suggestion to ensure transparency and reproducibility.

**Methods (Reviewer #1) – Study Design and Samples – Question 6:** The sample size estimation would benefit from additional clarification. Please specify the power used, whether the reported margin of error (1.7) corresponds to a half-width or full-width interval, and provide a reference for the formula applied. For context, estimating 99% sensitivity with a 3.5% full-width margin of error, 95% confidence, and 80% power requires approximately 254 confirmed cases (and a similar number of negative cases for 99% specificity), according to (Zhou, X. H., McClish, D. K., & Obuchowski, N. A. (2009). Statistical methods in diagnostic medicine. John Wiley & Sons). This may help explain the uncertainty observed, particularly for specificity.

**Reply:** We thank the reviewer for this insightful comment and for providing a valuable methodological reference. We have clarified the assumptions used for sample size estimation and now explicitly state that the reported margin of error corresponds to the half-width of the 95% confidence interval. We acknowledge that, although the number of *T. cruzi*–positive samples approached the estimated requirement, the number of negative samples was lower than that recommended for high-precision estimation of specificity. This likely contributed to the wider confidence intervals observed for specificity in our study. As this was a retrospective study based on available samples from a well-characterized biobank, sample size was constrained by sample availability rather than prospective recruitment. Despite this, the sample size allowed robust estimation of sensitivity and overall diagnostic performance. This limitation has now been explicitly acknowledged in the Discussion.

**Methods (Reviewer #1) – Study Design and Samples – Question 7:** Please clarify whether an intended sample size was defined for stratified analyses (by country and cardiac involvement), and how this was determined.

**Reply:** We thank the reviewer for this important comment. We clarify that no specific sample size was prospectively defined for stratified analyses by geographical origin or cardiac involvement. As this was a retrospective study based on samples from an existing biobank, the overall sample size was determined by sample availability rather than by predefined subgroup requirements. Stratified analyses were conducted as exploratory assessments to investigate the consistency of test performance across clinically and epidemiologically relevant subgroups. However, the study was not specifically powered for these subgroup comparisons. This limitation has now been explicitly acknowledged in the Discussion to avoid overinterpretation of stratified results.

**Methods (Reviewer #1) – Study Design and Samples – Question 8:** Please indicate whether any External Quality Assessment (EQA) was conducted. If not, a brief discussion of this point would still be informative. Use of a serological EQA panel aligned with international or national standards could strengthen confidence in test performance and observer consistency.

**Reply:** We thank the reviewer for this important comment. We clarify that no formal External Quality Assessment (EQA) scheme was conducted as part of this study. However, internal quality control measures were implemented to ensure the reliability of test execution and interpretation. All assays were performed in accordance with the manufacturer’s instructions, and results were independently interpreted by two blinded observers. In cases of uncertainty or disagreement, a third observer was consulted, and the test was repeated when consensus could not be reached. We acknowledge that the inclusion of a formal EQA panel aligned with international or national standards could further strengthen confidence in test performance and reproducibility. This point has now been acknowledged in the Discussion as a study limitation.

**Methods (Reviewer #1) – Study Design and Samples – Question 9:** Please clarify the blinding procedures applied in the study. Specifically, were observers blinded to clinical information, reference test results, and each other’s interpretations? This information is important for assessing the potential risk of review bias. If full blinding was not feasible, please discuss any potential impact on the findings.

**Reply:** We thank the reviewer for this important comment. We have clarified the blinding procedures in the Methods section. Specifically, the two primary readers were blinded to the reference test results and to any clinical or epidemiological information associated with the samples, and they interpreted the tests independently, without access to each other’s readings. In cases of uncertainty or disagreement, a third observer was consulted. The manuscript has been revised according to the reviewer’s suggestion to ensure transparency and reproducibility.

**Results (Reviewer #1) – Question 10:** Please consider adding p-values or clearly indicating whether comparisons are statistically significant, not only in the main text but also in the corresponding tables.

**Reply:** We thank the reviewer for this valuable suggestion. We have revised the Results section to explicitly indicate statistical significance and have also updated the corresponding tables to improve clarity and transparency. P-values derived from pairwise comparisons using Fisher’s exact test are now clearly reported in the text. In addition, a note has been added to the relevant tables to indicate that no statistically significant differences were observed. The manuscript has been revised according to the reviewer’s suggestion to ensure transparency and reproducibility.

**Results (Reviewer #1) – Question 11:** If available, please indicate whether the distribution of alternative diagnoses is known among samples classified as negative.

**Reply:** We thank the reviewer for this important comment. Information regarding alternative diagnoses among individuals classified as *T. cruzi*–negative was not available. Negative samples were defined according to the composite serological reference standard, but no additional clinical characterization of alternative diagnoses was available for this panel.

**Discussion (Reviewer #1) – Question 12:** Please clarify whether the test configuration evaluated (IBMP-8.1 and IBMP-8.4 antigens) corresponds to the same product version used in previous evaluations.

**Reply:** We thank the reviewer for this important question. The test evaluated in this study corresponds to the same commercial version of the TR Chagas Bio-Manguinhos assay that has been assessed in previous studies. The assay configuration, including the use of the IBMP-8.1 and IBMP-8.4 chimeric antigens, remains unchanged. The product is manufactured by Bio-Manguinhos/Fiocruz and supplied to the Brazilian Ministry of Health, with no modifications in its antigenic composition or test design relative to earlier evaluations. Therefore, the performance observed in this study is directly comparable to previously published data The manuscript has been revised according to the reviewer’s suggestion to ensure transparency and reproducibility.

**Discussion (Reviewer #1) – Question 13:** Additional discussion of study limitations, including potential sources of bias and statistical uncertainty, would strengthen interpretation of the findings, particularly regarding uncertainty in specificity estimates, the timing between index and reference testing, and possible effects of long-term sample storage.

**Reply:** We thank the reviewer for this important and constructive comment. The Discussion section has been revised to provide a more comprehensive and structured appraisal of study limitations and potential sources of bias. Specifically, we now explicitly address uncertainty in specificity estimates related to the number of negative samples, the retrospective nature of the study and the lack of control over the time interval between index and reference testing, and the potential impact of long-term sample storage on antibody stability. We also clarify limitations related to the absence of formal external quality assessment (EQA) and the availability of clinical and geographical data. These revisions improve the transparency and balance of the interpretation without altering the overall conclusions of the study. The manuscript has been revised according to the reviewer’s suggestion to ensure transparency and reproducibility.

**Discussion (Reviewer #1) – Question 14:** Discussion of the current regulatory status and use of the test (in Brazil only?), as well as its potential relevance for market entry in other countries, would add value. Given the unique position of Bio-Manguinhos as a public manufacturer in a fragmented market, this discussion could be particularly informative in the context of access in underserved populations and affordability.

**Reply:** We thank the reviewer for this insightful suggestion. We have expanded the Discussion to clarify the current regulatory status and use of the TR Chagas Bio-Manguinhos test, as well as its potential relevance for broader implementation. The assay is currently produced by Bio-Manguinhos/Fiocruz, a public manufacturer, and is used within the Brazilian public health system as part of national strategies for Chagas disease control. Although its current use is primarily within Brazil, the present findings, together with previous multicountry evaluations, support its potential applicability in other endemic settings. We also briefly discuss the relevance of public-sector manufacturing in facilitating access to diagnostic tools for neglected diseases, particularly in underserved populations, where affordability and supply stability remain critical challenges. The manuscript has been revised according to the reviewer’s suggestion to ensure transparency and reproducibility.

**Acknowledgements (Reviewer #1) – Question 15:** Please clarify the role of the test manufacturer/developer (Bio-Manguinhos) in the study. Specifically, indicate whether the tests were donated and whether the manufacturer had any role in study design, supervision, data analysis, or interpretation.

**Reply:** We thank the reviewer for this important comment. We have clarified the role of the manufacturer in the Acknowledgements section. The TR Chagas Bio-Manguinhos tests used in this study were provided by Bio-Manguinhos/Fiocruz. The manufacturer had no role in the study design, data collection, data analysis, interpretation of results, or decision to publish The manuscript has been revised according to the reviewer’s suggestion to ensure transparency and reproducibility.

**Editorial and Data Presentation Modifications (Reviewer #1) – Question 16:** Throughout the manuscript, please consider avoiding terms such as “chronic CD,” “CD screening” or “cases of CD” In many instances, more precise terminology would be “detection of chronic *T. cruzi* infection” “screening for *T. cruzi* infection” or “individuals infected with T. cruzi” as not all infected individuals develop clinical disease.

**Reply:** We thank the reviewer for this important comment. We have revised the manuscript to improve terminological precision and to better distinguish between infection and disease. Specifically, expressions such as “chronic CD,” “CD screening,” and “cases of CD” have been replaced, where appropriate, by more accurate terms such as “chronic *T. cruzi* infection,” “screening for *T. cruzi* infection,” and “individuals infected with *T. cruzi*.”

**Editorial and Data Presentation Modifications/Study Design and Samples (Reviewer #1) – Question 17:** Please clarify whether photographs of the evaluated index test were taken and whether they could be made available upon request.

**Reply:** We thank the reviewer for this suggestion. Photographic documentation of the index test was obtained during the study and can be made available upon reasonable request. All tests were performed and interpreted according to the manufacturer’s instructions under controlled laboratory conditions. The manuscript has been revised according to the reviewer’s suggestion to ensure transparency and reproducibility.

**Editorial and Data Presentation Modifications/Study Design and Samples (Reviewer #1) – Question 18:** Please clarify whether discordant interpretations resolved by a third observer were adjudicated directly from the cassette or using stored images.

**Reply:** We thank the reviewer for this important clarification. Discordant interpretations were adjudicated by a third observer through direct visual inspection of the original test cassette at the time of reading, rather than using stored images. This approach ensured consistency with routine diagnostic practice and minimized potential bias associated with image-based interpretation. The manuscript has been revised according to the reviewer’s suggestion to ensure transparency and reproducibility.

**Editorial and Data Presentation Modifications/Study Design and Samples (Reviewer #1) – Question 19:** If available, reporting inter-observer agreement and the rate of invalid test results would further strengthen the results.

**Reply:** We thank the reviewer for this valuable suggestion. Inter-observer agreement was high, with only one test requiring adjudication by a third observer. No invalid test results were observed. Although inter-observer agreement was not formally quantified using statistical measures such as Cohen’s kappa, the very low frequency of discordant interpretations supports the consistency of result interpretation. The manuscript has been revised according to the reviewer’s suggestion to ensure transparency and reproducibility.

**Editorial and Data Presentation Modifications/** **Discussion (Reviewer #1) – Question 20:** The paragraph spanning lines 298–322 could be shortened or divided into two parts to improve readability.

**Reply:** We thank the reviewer for this helpful suggestion. The paragraph has been revised to improve readability by dividing it into two parts and streamlining the presentation of ideas. These changes enhance clarity without altering the scientific content or interpretation.

**Editorial and Data Presentation Modifications/** **Discussion (Reviewer #1) – Question 21:** Please note that the study by Marchiol et al. is not a multicenter study; revising this wording would improve accuracy.

**Reply:** We thank the reviewer for this important correction. The wording has been revised to accurately reflect that the study by Marchiol et al. was not a multicenter study. This change improves the accuracy of the manuscript. The manuscript has been revised according to the reviewer’s suggestion to ensure transparency and reproducibility.

**Editorial and Data Presentation Modifications/** **Discussion (Reviewer #1) – Question 22:** Please specify which references are meant by “prior evaluations frequently included more heterogeneous negative panels”.

**Reply:** We thank the reviewer for this important comment. The statement has been revised to explicitly identify the studies supporting this observation. Specifically, the references by Marchiol et al. [8], Iturra et al. [7], and López et al. [9] have been incorporated to clarify that these prior evaluations included more heterogeneous negative panels. In addition, only the study by Iturra et al. [7] is cited in relation to the inclusion of samples from individuals with other infectious diseases associated with potential cross-reactivity, such as leishmaniasis, ensuring accurate representation of the literature. The manuscript has been revised according to the reviewer’s suggestion to ensure transparency and reproducibility.

**Editorial and Data Presentation Modifications/** **Discussion (Reviewer #1) – Question 23:** The discussion would benefit from addressing potential lot-to-lot variation. If available, please indicate whether this aspect was considered in the present study or reported in previous evaluations of the index test.

**Reply:** We thank the reviewer for this important point. Lot-to-lot variation was not specifically evaluated in the present study, and information regarding the specific production lots used in previous studies is generally not reported in the literature. However, the TR Chagas Bio-Manguinhos test is a commercially registered product manufactured under Good Manufacturing Practices (GMP) and subject to regulatory oversight by the Brazilian Health Regulatory Agency (ANVISA), which includes quality control procedures to ensure batch consistency. A statement acknowledging this aspect has been included in the Discussion.

**Summary and General Comments (Reviewer #1) – Question 23:** It is a very interesting and well-structured study about a novel evaluation of the index test with samples from the Southern Cone. I consider it makes valuable contributions to the field of *Trypanosoma cruzi* infection detection. The work will be of interest to researchers, test developers and manufacturers, practitioners, and policy makers in the field of NTDs, as well as to broader audiences. The study is methodologically robust, with clearly reported findings supported by appropriate analytical approaches, and was conducted in accordance with ethical requirements. There are a few minor issues that should be addressed to improve the clarity and robustness of the manuscript. Therefore, I recommend the manuscript for publication pending minor revisions, which are detailed above. I hope this is helpful.

**Reply:** We sincerely thank the reviewer for the careful reading of our manuscript and for the constructive and encouraging comments. We greatly appreciate the recognition of the relevance and methodological robustness of our study. The reviewer’s suggestions were very helpful and substantially improved the clarity, precision, and overall quality of the manuscript. We are grateful for these valuable contributions.

**Reviewer #2**

**Methods/Study design and samples (Reviewer #2) – Question 01:** How was *T. cruzi* infection status defined? How many assays were used?

**Reply:** We thank the reviewer for this important comment. *T. cruzi* infection status was defined based on the original serological results obtained at the participating institutions using commercial assays routinely employed for the diagnosis of chronic *T. cruzi* infection, in accordance with national and international recommendations. At the Instituto Nacional de Parasitología “Dr. Mario Fatala Chabén”, the diagnostic workup included two assays: CMIA Architect Chagas (ARCHITECT Chagas®, Abbott, Illinois, USA) and Chagatest ELISA recombinante v.4.0 (Wiener lab®, Rosario, Argentina). At the Hospital General de Agudos “Dr. Ignacio Pirovano”, routine diagnosis included two assays: Chagatest HAI (Wiener lab®, Rosario, Argentina) and CMIA on the Alinity i platform (Abbott, Illinois, USA). In cases of discrepancy at Pirovano, an additional hemagglutination assay (SERODIA-CHAGAS, Fujirebio Inc., Tokyo, Japan) was used in routine practice; however, discordant cases were not included in the sample panel analyzed in the present study. Thus, the samples included in this study were classified based on concordant serological results from two assays performed in the source institutions. We have clarified this point in the Methods section.

**Methods/Study design and samples (Reviewer #2) – Question 02** Were samples selected consecutively or convenience samples from the repository? Could spectrum bias affect test performance estimates?

**Reply:** We thank the reviewer for this important comment. The samples used in this study were selected as convenience samples from the INGEBI-CONICET repository rather than consecutively enrolled. As this was a retrospective study based on pre-existing, well-characterized samples, selection was determined by sample availability and eligibility criteria rather than by prospective consecutive recruitment. We acknowledge that this sampling strategy may introduce spectrum bias and could influence diagnostic performance estimates. This limitation has now been explicitly acknowledged in the Discussion section.

**Methods/Study design and samples (Reviewer #2) – Question 03:** Line 133: Any samples with discordant results? PCR test performed.

**Reply:** We thank the reviewer for this important comment. In cases of discordant serological results, classification was resolved using a third serological assay (hemagglutination assay: SERODIA-CHAGAS, Fujirebio Inc., Tokyo, Japan) rather than PCR. This approach is consistent with international recommendations for the diagnosis of chronic *T. cruzi* infection, in which molecular methods are not recommended as confirmatory tools because of their limited sensitivity in the chronic phase.

**Methods/Study design and samples (Reviewer #2) – Question 04:** Line 140: Please specify which third confirmatory assay was used to resolve discordant results (manufacturer, platform, antigen target, cutoff criteria).

**Reply:** We thank the reviewer for this important comment. In the routine diagnostic workflow at the Hospital General de Agudos “Dr. Ignacio Pirovano”, discrepant serological results were resolved using an additional hemagglutination assay, SERODIA-CHAGAS (Fujirebio Inc., Tokyo, Japan). However, samples with discordant results were not included in the panel analyzed in the present study. We have clarified this point in the Methods section. Because these discordant cases were excluded from the study sample, the third assay was not part of the final classification algorithm applied to the panel analyzed here.

**Methods/Study design and samples (Reviewer #2) – Question 05:** How many samples showed discordant results between the index test and the reference standard?

**Reply:** We thank the reviewer for this important comment. A total of 11 samples showed discordant results between the index test and the reference standard. Specifically, 7 samples classified as T. cruzi–positive by the reference standard were non-reactive by the TR Chagas Bio-Manguinhos assay, and 4 samples classified as negative by the reference standard were reactive by the index test. This information is explicitly reflected in the diagnostic performance calculations presented in the Results section, as well as in Figure 1.

**Methods/Study design and samples (Reviewer #2) – Question 06:** Were discordant samples reclassified based solely on the third test, or using a composite algorithm?

**Reply:** We thank the reviewer for this important comment. Samples with discordant serological results in the source institutions were not reclassified for inclusion in the present study. Instead, these cases were excluded from the sample panel analyzed here. Therefore, the final study panel was composed only of samples with concordant serological classification in the participating institutions, and no reclassification algorithm based on a third test was applied within the analyzed dataset. We have clarified this point in the Methods section to avoid ambiguity.

**Methods/Study design and samples (Reviewer #2) – Question 07:** The supplementary table currently reports only final positive/negative serological profiles. Could the authors include a breakdown showing discordant cases and how they were resolved?

**Reply:** We thank the reviewer for this important comment. Samples with discordant serological classification according to the reference assays used at the source institutions were not included in the study panel analyzed here. Therefore, the supplementary table reports only the final concordant positive or negative serological profiles of the samples included in the study. We have clarified this point in the Methods section to avoid ambiguity.

**Methods/Reference standard (Reviewer #2) – Question 08:** The reference standard used to classify samples as *T. cruzi*–positive or negative is not clearly and explicitly defined. The specific assays included in the composite reference standard and the decision algorithm applied should be described in detail.

**Reply:** We thank the reviewer for this important comment. In the routine diagnostic workflow at the Hospital General de Agudos “Dr. Ignacio Pirovano”, discrepant serological results were resolved using an additional hemagglutination assay, SERODIA-CHAGAS (Fujirebio Inc., Tokyo, Japan). However, samples with discordant results were not included in the panel analyzed in the present study. We have clarified this point in the Methods section. Because these discordant cases were excluded from the study sample, the third assay was not part of the final classification algorithm applied to the panel analyzed here. The manuscript has been revised according to the reviewer’s suggestion to ensure transparency and reproducibility.

**Methods/Handling of discordant results (Reviewer #2) – Question 09:** The Methods state that discordant serological results were resolved using a third confirmatory test; however, the specific assay used, the number of discordant samples, and how these cases were incorporated into the final analysis are not reported.

**Reply:** We thank the reviewer for this important comment. We have clarified this point in the revised Methods section. In the routine diagnostic workflow at the Hospital General de Agudos “Dr. Ignacio Pirovano”, discrepant serological results were resolved using an additional hemagglutination assay, SERODIA-CHAGAS (Fujirebio Inc., Tokyo, Japan). However, samples with discordant serological classification at the source institutions were not included in the panel analyzed in the present study. Therefore, no discordant cases were incorporated into the final study sample, and the panel analyzed here consisted only of samples with concordant serological classification according to the reference assays used at the participating institutions. This point is now explicitly stated in the Methods section to avoid ambiguity.

**Methods/Antigen composition of the test (Reviewer #2) – Question 10:** The antigenic composition of the TR Chagas Bio-Manguinhos rapid test is not described in the Methods section and is only mentioned in the discussion. For transparency and reproducibility, this information should be clearly stated in the Methods.

**Reply:** We thank the reviewer for this important comment. We agree that antigenic composition should be clearly stated in the Methods section for transparency and reproducibility. Although the antigenic composition of many commercial rapid diagnostic tests is not publicly disclosed, this information is available for the TR Chagas Bio-Manguinhos assay was already described in the Methods section of the version submitted for review. Specifically, the manuscript states that the device incorporates two recombinant chimeric antigens, IBMP-8.1 and IBMP-8.4. For clarity, the relevant sentence is reproduced below. We hope this clarification resolves the reviewer’s concern.

“The index test was the TR Chagas Bio-Manguinhos (Bio-Manguinhos/Fiocruz, Rio de Janeiro, Brazil), a lateral-flow immunochromatographic assay for the qualitative detection of anti-T. cruzi IgG. The device incorporates two recombinant chimeric antigens (IBMP-8.1 and IBMP-8.4) and an internal control line immobilized on a multimembrane strip assembled under an adhesive card to permit capillary flow [11].”

**Methods/Sample size and subgroup analyses (Reviewer #2) – Question 11:** While sample size calculations are provided, additional clarification is needed regarding the assumptions used and how the final sample size relates to the stratified analyses by geography and clinical stage.

**Reply:** We thank the reviewer for this important comment. We have clarified the assumptions underlying the sample size calculation in the Methods section. Specifically, sample size was “estimated assuming a 1.7% margin of error (half-width of the 95% confidence interval), expected sensitivity of 99%, specificity of 99.5%, and a 95% confidence level, based on standard formulas for proportions in diagnostic accuracy studies [12].” As this was a retrospective study based on samples from an existing repository, the final sample size was determined by sample availability rather than prospective recruitment. The resulting sample size was adequate for overall diagnostic performance estimation, particularly for sensitivity, but no specific sample size was prospectively defined for stratified analyses by geographical origin or Kuschnir cardiac stage. Therefore, these subgroup analyses should be interpreted as exploratory and with appropriate caution. This point has now been clarified in both the Methods and Discussion sections.

**Methods/Sample selection and representativeness (Reviewer #2) – Question 12:** Additional details on sample selection, geographical coverage, and clinical spectrum would help contextualize the findings, particularly given the use of archived plasma samples and the absence of province-level data.

**Reply:** We thank the reviewer for this important comment. We have expanded the Methods and Discussion sections to better contextualize sample selection, geographical coverage, and clinical spectrum. Specifically, we now clarify that archived plasma samples were selected as convenience samples from the INGEBI-CONICET repository according to availability and eligibility criteria. We also state more explicitly that, although participants originated from Argentina, Bolivia, and Paraguay, province- or city-level information was not available, and all individuals resided in Buenos Aires at the time of clinical evaluation. In addition, the clinical spectrum of the positive group is now better defined through the description of Kuschnir cardiac stages, while also acknowledging that information on gastrointestinal involvement was not available. These additions were made to improve the epidemiological and clinical interpretation of the findings while making clear the limitations inherent to the retrospective use of archived samples.

**Methods/Statistical analysis (Reviewer #2) – Question 13:** The statistical methods are generally appropriate; however, the limited power of stratified analyses, as mentioned, should be acknowledged when interpreting subgroup comparisons.

**Reply:** We thank the reviewer for this important comment. We agree that the stratified analyses were not specifically powered for subgroup comparisons and should therefore be interpreted with caution. As this was a retrospective study based on available repository samples, no prospective sample size was defined for analyses by geographical origin or Kuschnir cardiac stage. This limitation has now been explicitly acknowledged in the Discussion to avoid overinterpretation of subgroup findings.

**Results (Reviewer #2) – Question 14:** Although this study is presented as the first evaluation of this assay in this region, the manuscript does not describe the antigenic targets or peptide composition of the test. Additional details on antigen composition would help readers place this assay within the broader landscape of existing Chagas serological tests.

**Reply:** We thank the reviewer for this comment. This point was addressed in our response to Reviewer #2, Question 10. As clarified there, the antigenic composition of the TR Chagas Bio-Manguinhos assay was already described in the Methods section of the version submitted for review. Specifically, the manuscript states that the device incorporates two recombinant chimeric antigens, IBMP-8.1 and IBMP-8.4. For clarity, the relevant sentence is reproduced below. We hope this clarification resolves the reviewer’s concern.

“The index test was the TR Chagas Bio-Manguinhos (Bio-Manguinhos/Fiocruz, Rio de Janeiro, Brazil), a lateral-flow immunochromatographic assay for the qualitative detection of anti-T. cruzi IgG. The device incorporates two recombinant chimeric antigens (IBMP-8.1 and IBMP-8.4) and an internal control line immobilized on a multimembrane strip assembled under an adhesive card to permit capillary flow [11].”

**Results (Reviewer #2) – Question 15:** Table 1. The authors state that diagnostic performance was evaluated against a reference standard; however, Table 1 does not specify which reference standard was used. Because sensitivity, specificity, likelihood ratios, and agreement metrics are entirely dependent on the reference standard, this information is essential for interpretation and reproducibility. Please clearly identify the reference standard used (including assays and decision algorithm) in both the Methods and Table 1.

**Reply:** We thank the reviewer for this important comment. We agree that the reference standard must be clearly identified for proper interpretation of the diagnostic performance estimates. In the revised Methods section, we now specify that the reference classification was based on the original concordant serological results obtained at the participating institutions using commercial assays routinely employed for the diagnosis of chronic T. cruzi infection. At the Instituto Nacional de Parasitología “Dr. Mario Fatala Chabén”, the assays used were CMIA Architect Chagas (ARCHITECT Chagas®, Abbott, Illinois, USA) and Chagatest ELISA recombinante v.4.0 (Wiener lab®, Rosario, Argentina). At the Hospital General de Agudos “Dr. Ignacio Pirovano”, the assays used were Chagatest HAI (Wiener lab®, Rosario, Argentina) and CMIA on the Alinity i platform (Abbott, Illinois, USA). Only samples with concordant serological classification in the source institutions were included in the study panel. To improve clarity and reproducibility, we also revised Study design and samples subsection to explicitly indicate that diagnostic performance was evaluated against this composite serological reference standard.

**Results (Reviewer #2) – Question 16:** Table 2. The authors state that “all 21 Paraguayan-born individuals with confirmed infection were correctly classified, resulting in a sensitivity of 100% (95% CI: 84.5%–100%).” However, in Table 2, the total number of Paraguayan-born individuals is reported as n=8, which is inconsistent with the reported number of true positives (TP=21). Please revise Table 2 and ensure that the total number of samples, true positives, and false negatives are correctly and consistently reported.

**Reply:** We thank the reviewer for carefully identifying this inconsistency. The value reported for the Paraguayan subgroup in Table 2 was incorrect due to a typographical error. The correct total number of *T. cruzi*–positive Paraguayan-born individuals is 21, not 8. Table 2 has now been corrected accordingly, and the values for total sample number, true positives, false negatives, and sensitivity are now internally consistent.

**Results (Reviewer #2) – Question 17:** Iine 269: The number of samples in advanced stages, particularly stage 2 (n=8) is very small, resulting in wide confidence intervals and limited statistical power. Therefore, the absence of statistically significant differences is more likely due to limited sample size than true equivalence of sensitivity across stages. From a serological standpoint, this analysis should be viewed as exploratory or taken with caution.

**Reply:** We thank the reviewer for this important comment. We agree that the limited number of samples in advanced Kuschnir stages, particularly stage 2, resulted in wide confidence intervals and reduced statistical power for subgroup comparisons. Accordingly, the absence of statistically significant differences should not be interpreted as evidence of equivalence of sensitivity across stages. We have revised the manuscript to clarify that the stratified analysis by cardiac involvement should be interpreted as exploratory and with caution. The manuscript has been revised according to the reviewer’s suggestion to ensure transparency and reproducibility.

**Conclusions (Reviewer #2) – Question 18:** Line 296: It is unclear how the authors can conclude that the TR Chagas Bio-Manguinhos test "matched or exceeded" the sensitivity of other LFAs evaluated in multicountry comparisons, as this study did not include samples from a wide range of countries with known differences in circulating DTUs or parasite strains, nor direct head-to-head comparisons.

Reply: We thank the reviewer for this important comment. We agree that the wording was too strong, as the present study did not include direct head-to-head comparisons with other LFAs, nor samples from a broader range of countries with different circulating DTUs. We have therefore revised the Discussion to moderate this interpretation. The statement no longer suggests that the assay “matched or exceeded” the sensitivity of other LFAs, and now more appropriately indicates that the diagnostic performance observed in this study falls within the upper range of values reported in previous evaluations. The manuscript has been revised

**Conclusions (Reviewer #2) – Question 19:** Line 331: Given the limited sample sizes and wide confidence intervals in several stratified analyses. Please clarify.

**Reply:** We thank the reviewer for this important comment. We agree that the limited sample sizes and wide confidence intervals in some stratified analyses, particularly in the advanced Kuschnir stages and in smaller geographical subgroups, require cautious interpretation. We have therefore revised the Discussion and Conclusion to avoid overinterpretation of subgroup findings and to make clear that these analyses should be considered exploratory. The manuscript has been revised according to the reviewer’s suggestion to ensure transparency and reproducibility.

**Conclusions/Major comment (Reviewer #2) – Question 20:** The manuscript cites prior work using IBMP-based chimeric antigens; however, the antigenic composition of the TR Chagas Bio-Manguinhos assay evaluated here is not clearly described. Neither the present study nor the referenced work provides sufficient detail regarding the specific antigens or peptides/epitopes included in the test. The description of antigen composition would facilitate interpretation of the biological basis of the assay and help readers better contextualize this platform relative to existing commercial tests (e,g., Wiener, InBios). Given that this is presented as the first evaluation of the assay in this region, such clarification is important to support interpretation of the findings.

**Reply:** We thank the reviewer for this important comment. We agree that clearer description of the antigenic basis of the assay helps contextualize the platform within the broader landscape of Chagas serological tests. In the present study, the antigenic composition that can be explicitly described is that the TR Chagas Bio-Manguinhos assay incorporates two recombinant chimeric antigens, IBMP-8.1 and IBMP-8.4, as stated in the Methods section. More detailed information at the level of specific peptides or individual epitopes included in the commercial device is publicly available. To improve transparency, we have clarified this point in the Discussion.

**Editorial and Data Presentation Modifications (Reviewer #2) – Question 21:** The manuscript will benefit from several editorial and presentation related clarification to improve methods. In particular, the antigens used in each diagnostic assay should be explicitly stated in the Methods section and clearly linked to the corresponding results and figures, rather than being implied. Additionally, clearer descriptions of the reference standard used for test evaluation and more explicit labeling of tables and figures would improve interpretability.

**Reply:** We thank the reviewer for this helpful comment. We agree that clearer presentation of the index test and reference standard improves the interpretability of the manuscript. In the revised version, we have clarified in the Methods section that the TR Chagas Bio-Manguinhos assay incorporates the recombinant chimeric antigens IBMP-8.1 and IBMP-8.4. We have also revised the description of the reference standard to state more explicitly that infection status was defined using a composite serological reference standard based on concordant results from at least two independent assays employing different methodological principles, with discordant results resolved by a third serological assay. In addition, we have improved the wording of the corresponding table titles and legends to make clear that diagnostic performance was evaluated against a composite serological reference standard, thereby improving the linkage between the Methods, Results, and tables.

**Summary and General Comments (Reviewer #2) – Question 22:** The study addresses an important regional gap in Chagas diagnostics and provides useful data from an underrepresented setting. However, major revisions are required to clarify key methodological aspects, including antigen composition, definition of the reference standard, and handling of discordant samples. In addition, the interpretation of stratified analyses should be moderated, and the Discussion should be more closely aligned with the data generated in this study, particularly with respect to claims of novelty and comparative performance with existing commercial assays.

**Reply:** We sincerely thank the reviewer for the careful reading of our manuscript and for the constructive and insightful comments. We greatly appreciate the recognition of the relevance of the study and its contribution to an underrepresented regional setting. The reviewer’s observations were very helpful and allowed us to improve key methodological clarifications, moderate the interpretation of stratified analyses, and better align the Discussion with the data generated in this study. We are grateful for these valuable contributions, which have substantially strengthened the manuscript.

**Reviewer #3**

**Methods/Study design and samples (Reviewer #3) – Question 01:** Although the Introduction states that “In this context, we evaluated the diagnostic performance of the TR Chagas Bio‑Manguinhos test using serum samples from Argentina, Bolivia, and Paraguay, applying international guidelines for diagnostic test validation,” the subsequent Methods and Results sections introduce additional analyses related to the country of origin of the samples and the cardiological involvement of the patients. These important analytical dimensions are not mentioned or anticipated in the Introduction. For coherence and to guide the reader, the Introduction should briefly indicate that the study also examines potential differences by sample origin and by clinical/cardiovascular status.

**Reply:** We thank the reviewer for this important comment. We agree that the Introduction should better anticipate the additional subgroup analyses presented in the Methods and Results sections. To improve coherence and guide the reader more clearly, we have revised the final sentence of the Introduction to indicate that the study also explored diagnostic performance according to country of origin and cardiac involvement. The manuscript has been revised according to the reviewer’s suggestion to ensure transparency and reproducibility.

**Methods/Study design and samples (Reviewer #3) – Question 02:** The study design is appropriate, and the target population is well defined. Ethical approvals are adequate. The analytical tools employed are suitable. However, the authors do not clearly demonstrate whether the sample size is statistically powered to support stratified analyses based on country of origin or cardiological involvement.

**Reply:** We thank the reviewer for this important comment. We agree that the study was not specifically powered to support stratified analyses by country of origin or cardiological involvement. As this was a retrospective study based on samples from an existing repository, the final sample size was determined by sample availability rather than by predefined subgroup requirements. We have clarified in the Methods section that no specific sample size was prospectively defined for these subgroup analyses, and we now explicitly acknowledge in the Discussion that these comparisons should be interpreted as exploratory and with caution. The manuscript has been revised according to the reviewer’s suggestion to ensure transparency and reproducibility.

**Methods/Study design and samples (Reviewer #3) – Question 03:** Line 129. For readers’ comprehension, please state explicitly that the study is retrospective and observational, and that samples were obtained from an INGEBI-CONICET serotheque/biorepository?

**Reply:** We thank the reviewer for this helpful comment. We have revised the Methods section to state more explicitly that this was a retrospective observational study and that the samples were obtained from the INGEBI-CONICET repository. The manuscript has been revised according to the reviewer’s suggestion to ensure transparency and reproducibility.

**Methods/** **Sample size and stratification (Reviewer #3) – Question 04:** Line 133. In addition to describing the sample size calculation, please specify whether the resulting N supports stratified analyses by country of origin and by clinical/cardiovascular classification. Include the biostatistical approach and bibliographic reference for calculating sample size.

**Reply:** We thank the reviewer for this important comment. We have clarified the sample size calculation in the Methods section, including the underlying assumptions, the biostatistical approach, and the bibliographic reference used. Specifically, sample size was estimated based on standard formulas for proportions in diagnostic accuracy studies, assuming a 1.7% margin of error (half-width of the 95% confidence interval), expected sensitivity of 99%, specificity of 99.5%, and a 95% confidence level. We also clarify that the resulting sample size was intended to support overall diagnostic performance estimation and that no specific sample size was prospectively defined for stratified analyses by country of origin or cardiac/cardiovascular classification. Therefore, these subgroup analyses should be interpreted as exploratory and with caution. The manuscript has been revised according to the reviewer’s suggestion to ensure transparency and reproducibility.

**Methods/Not multicenter (Reviewer #3) – Question 05:** Line 134. For clarity, please state that although samples originate from multiple countries, this is not a multicenter study; it is a single center evaluation using serum samples from people from three countries.

**Reply:** We thank the reviewer for this important clarification. We agree that the study should not be described as multicenter. Although the samples originated from individuals from three countries, this was a single-center evaluation based on plasma samples analyzed in a single study setting. We have revised the Methods section accordingly to avoid ambiguity. The manuscript has been revised according to the reviewer’s suggestion to ensure transparency and reproducibility.

**Methods/Clinical evaluation and sampling (Reviewer #3) – Question 06:** Line 136. The text mentions medical evaluation. Because this is an RDT performance assessment, please also describe the sample collection process (collection setting and procedures) in addition to clinical evaluation.

**Reply:** We thank the reviewer for this important comment. We have revised the Methods section to better describe the sample collection process in addition to the clinical evaluation. Specifically, we now clarify that samples were obtained from individuals evaluated at the Instituto Nacional de Parasitología “Dr. Mario Fatala Chabén” and at the Hospital General de Agudos “Dr. Ignacio Pirovano” in Buenos Aires, Argentina, either during routine clinical evaluation or during scheduled blood collection visits requested as part of diagnostic workup. Blood samples were collected in the participating institutions and processed locally to obtain plasma, which was subsequently archived for repository use. The manuscript has been revised according to the reviewer’s suggestion to ensure transparency and reproducibility.

**Methods/Sample metadata and matrix (Reviewer #3) – Question 07:** Line 137. In the Samples section, please include: dates of collection, storage conditions (temperature, duration, freeze–thaw history), and explicitly state the biological matrix (plasma).

**Reply:** We thank the reviewer for this important comment. We have revised the Samples section to explicitly state the dates of collection, the biological matrix used, and the storage conditions of the archived material. Specifically, we now indicate that the study used plasma samples collected between 2015 and 2024. Plasma aliquots were stored at –20 °C in multiple tubes to minimize repeated freeze–thaw cycles. Because this was a retrospective study based on archived material collected over several years, storage duration varied according to the date of collection. This information has now been added to the Methods section to improve clarity and reproducibility.

**Methods/RDT reading procedures (Reviewer #3) – Question 08:** Please detail the reading procedures for RDT: • Number of operators (one vs. two readers; if discordant, was there a third adjudicator?). • Blinding: confirm that operators/readers were blinded to conventional serology results and any clinical data. • Training/competency of operators and any standardized reading time window and lighting conditions, if applicable.

**Reply:** We thank the reviewer for this important comment. We have revised the Methods section to provide a more detailed description of the RDT reading procedures. Briefly, all tests were independently interpreted by two readers. In cases of uncertainty or disagreement, a third observer adjudicated the result through direct visual inspection of the original test cassette. The readers were blinded to the conventional serological results and to any clinical or epidemiological information associated with the samples, and they performed their interpretations independently, without access to each other’s readings. Test results were read at the manufacturer-recommended time point of 15 minutes and under routine laboratory conditions. All readings were performed by laboratory personnel following the manufacturer’s instructions. The manuscript has been revised according to the reviewer’s suggestion to ensure transparency and reproducibility.

**Results/Stratified analyses feasibility (Reviewer #3) – Question 09:** Lines 266–269. Please clarify whether the sample size supports stratified analyses for the outcomes reported (e.g., by country of origin and by clinical/cardiovascular involvement), and present these analyses if adequately powered—or justify why not.

**Reply:** We thank the reviewer for this important comment. We clarify that the sample size was not specifically designed to support stratified analyses by country of origin or by cardiac/cardiovascular involvement. As this was a retrospective study based on samples from an existing repository, the final sample size was determined by sample availability rather than by predefined subgroup requirements. The stratified analyses were nevertheless performed as exploratory assessments to investigate whether test sensitivity varied across clinically and epidemiologically relevant subgroups. We have now made this limitation explicit in both the Methods and Discussion sections, and these subgroup findings should therefore be interpreted with caution. The manuscript has been revised according to the reviewer’s suggestion to ensure transparency and reproducibility.

**Results/Specificity analysis (Reviewer #3) – Question 10:** Line 235 onwards. Please explain why specificity was not analyzed and only sensitivity was reported in geographical origin and cardiac involvement. Adjust it for a complete analysis.

**Reply:** We thank the reviewer for this important comment. Specificity was not stratified by geographical origin or cardiac involvement because all negative samples in the study originated from Argentina. Therefore, subgroup-specific estimation of specificity was not possible. For this reason, only sensitivity was reported in the stratified analyses, as these subgroups were defined within the positive sample set. We have clarified this point in the Results section to improve transparency and avoid misinterpretation. The manuscript has been revised according to the reviewer’s suggestion to ensure transparency and reproducibility.

**Conclusions/Not multicenter (clarification needed) (Reviewer #3) – Question 11:** 1. Line 276. To avoid any misunderstanding, please state explicitly that this is not a multicenter study, but rather a single‑center evaluation using samples from patients originating from three countries.

**Reply:** We thank the reviewer for this important clarification. We agree that the study should not be interpreted as multicenter. Although the samples originated from individuals from three countries, this was a single-center evaluation based on plasma samples analyzed in Buenos Aires, Argentina. We have revised the Methods section accordingly to avoid any misunderstanding.

**Conclusions/Characterization of negatives in retrospective panels (Reviewer #3) – Question 12:** Line 315. In retrospective evaluations using pre‑characterized panels, negative samples are expected to be true negatives according to the reference standard. Therefore, statements suggesting possible cross‑reactivity with other infections (e.g., leishmaniasis) should be reconsidered or justified based on the actual composition and characterization of the negative panel used in this study. A revision of this point is recommended.

**Reply:** We thank the reviewer for this important comment. We agree that, in a retrospective evaluation using a pre-characterized serological panel, negative samples are expected to be true negatives according to the reference standard, and that possible cross-reactivity with other infections cannot be inferred unless the composition of the negative panel is explicitly known. We have therefore revised the Discussion to remove speculative wording suggesting possible cross-reactivity within the negative panel used in the present study. The revised text now focuses more appropriately on differences in panel composition and study design across evaluations. The manuscript has been revised according to the reviewer’s suggestion to ensure transparency and reproducibility.

**Conclusions/Mischaracterization of the cited study (Reviewer #3) – Question 13:** Line 300. The cited study is not multicenter; it was retrospective (using biobank/serotheque sera) and carried out under controlled laboratory conditions. The description should be corrected to accurately reflect the study design.

**Reply:** We thank the reviewer for this important correction. We agree that the cited study should not be described as multicenter. The wording has been revised to more accurately reflect that it was a retrospective, laboratory-based evaluation using archived sera under controlled conditions. The manuscript has been revised according to the reviewer’s suggestion to ensure transparency and reproducibility.

**Conclusions/Both sensitivity and specificity are context‑dependent (Reviewer #3) – Question 14:** Lines 315–317. The discussion currently frames specificity as the parameter conditioned by context; however, both sensitivity and specificity depend on multiple contextual factors, including Characteristics of the positive samples (e.g., chronic indeterminate Chagas with lower antibody titers may reduce sensitivity in some assays). Population‑level factors (e.g., immunosuppression, which can lead to diminished antibody production and thus lower serological sensitivity). Strain diversity/DTUs, given that antigenic variation may influence recognition. The reference standard used, as imperfect comparators, may bias estimates of both sensitivity and specificity.

Reply: We thank the reviewer for this important comment. We agree that both sensitivity and specificity are context-dependent parameters and may be influenced by multiple factors, including the clinical and immunological characteristics of the positive samples, population-level factors affecting antibody responses, parasite genetic diversity, and the composition of the reference standard used for classification. We have revised the Discussion accordingly to avoid framing specificity alone as context-dependent and to provide a more balanced interpretation of diagnostic performance. The manuscript has been revised according to the reviewer’s suggestion to ensure transparency and reproducibility.

**Conclusions (Reviewer #3) – Question 15:** In the Bolivian study by López et al. (2024)( López R, García A, Chura Aruni JJ, Balboa V, Rodríguez A, Erkosar B, et al. Comparative evaluation of lateral flow assays to diagnose chronic Trypanosoma cruzi infection in Bolivia. PLoS Negl Trop Dis 18(3): e0012016 ), sensitivity values were similar to those reported here; however, specificity differed markedly despite the substantially larger sample size (N = 398; positives = 216). The authors should explore and discuss potential reasons underlying this discrepancy, considering methodological differences, sample composition, and epidemiological context.

**Reply:** We thank the reviewer for this important comment. We agree that the discrepancy in specificity between the present study and the Bolivian evaluation by López et al. [9] warrants a more explicit discussion. Although sensitivity values were similar, differences in specificity may plausibly reflect methodological and contextual factors rather than intrinsic differences in assay behavior alone. These include differences in negative panel composition, study design, and epidemiological setting. In particular, prior evaluations such as López et al. [9] included more heterogeneous sample panels and were conducted in distinct endemic contexts, which may have influenced specificity estimates. We have revised the Discussion to make this comparison more explicit and to better contextualize the observed discrepancy.

**Conclusions/Sample size considerations and discrepancies with prior evaluations (Reviewer #3) – Question 16:** Previous evaluations in Bolivia and Colombia—where this RDT was assessed alongside several others—reported much higher specificity for the TR Bio‑Manguinhos test, using sample sizes comparable to those used for the other assays in those studies. Because those evaluations included a higher proportion of negative samples, the authors should clarify how the sample size assumptions used here address these differences and assess whether variation in sample composition may explain the lower specificity reported in the present study.

**Reply:** We thank the reviewer for this important comment. We agree that the interpretation of specificity should take into account both the sample size assumptions used in the present study and differences in sample composition relative to previous evaluations. As clarified in the revised Methods and Discussion, the sample size calculation was designed for overall diagnostic performance estimation, but the number of negative samples was lower than that required for high-precision estimation of specificity, which likely contributed to the wider confidence intervals observed for this parameter. In addition, differences in negative panel composition across studies may also have influenced specificity estimates. Previous evaluations conducted in Bolivia and Colombia included larger and more heterogeneous negative sample sets, which may partly explain discrepancies relative to the present study. We have revised the Discussion to make these points more explicit and to avoid overinterpretation of cross-study comparisons.

**Conclusions/Implications for large‑scale implementation and cost‑effectiveness (Reviewer #3) – Question 17:** Given the potential large‑scale use of rapid diagnostic tests for screening, assays with high sensitivity but more limited specificity could substantially increase downstream diagnostic confirmation costs. The authors should consider recommending cost‑effectiveness analyses to evaluate the feasibility of deploying this RDT in broad screening strategies.

**Reply:** We thank the reviewer for this important and forward-looking comment. We agree that, in large-scale screening strategies, even modest reductions in specificity may increase the number of individuals requiring confirmatory testing, with consequent effects on programmatic costs and operational feasibility. At the same time, screening strategies must also consider the consequences of imperfect sensitivity, since false-negative results may delay diagnosis and treatment and potentially increase downstream clinical and public health costs associated with undetected infection. Although a formal cost-effectiveness analysis was beyond the scope of the present diagnostic accuracy study, we agree that this is an important area for future research. Such evaluations should consider not only the cost of additional confirmatory testing generated by reactive results, but also the potential costs of missed diagnoses and delayed care in individuals who remain undetected. We have revised the Discussion accordingly.

**Conclusions/Need for discussion on confirmatory use and public health impact (Reviewer #3) – Question 18:** The Discussion section does not explore future directions for applied research regarding the potential use of RDTs as confirmatory diagnostic tools, nor does it address the broader public health implications should such applications prove viable. Expanding this section would significantly strengthen the translational and programmatic relevance of the manuscript.

**Reply:** We thank the reviewer for this important and constructive comment. We agree that discussion of future applied research directions could strengthen the translational relevance of the manuscript. Although the present study evaluated the TR Chagas Bio-Manguinhos assay as a screening tool and not as a confirmatory diagnostic test, we agree that future studies should explore whether high-performing RDTs might contribute to simplified diagnostic pathways, particularly in underserved settings where access to conventional laboratory methods is limited. If supported by additional evidence and regulatory evaluation, such applications could have important public health implications by facilitating earlier diagnosis and expanding access to care. We have revised the Discussion accordingly.

**Summary and General Comments (Reviewer #3) – Question 19:** The topic addressed in this manuscript is of substantial importance for advancing the simplification of diagnostic approaches for Chagas disease. The authors successfully highlight the need to expand the use of rapid diagnostic tests while simultaneously ensuring rigorous evaluation of their quality. This is a highly relevant and timely contribution to the field, and the manuscript is therefore of considerable interest.

**Reply:** We sincerely thank the reviewer for the careful reading of our manuscript and for the thoughtful and encouraging comments. We greatly appreciate the recognition of the relevance and timeliness of our study. The reviewer’s expertise and experience have contributed substantially to improving the clarity, rigor, and overall quality of the manuscript. We are very grateful for these valuable contributions.
